# Development of a bioassay method for larval western corn rootworm (Coleoptera: Chrysomelidae)

**DOI:** 10.1093/jee/toaf202

**Published:** 2025-09-03

**Authors:** Abigail L Kropf, Ethan Goes, Aaron J Gassmann

**Affiliations:** Department of Plant Pathology, Entomology and Microbiology, Iowa State University, Ames, IA, USA; Department of Plant Pathology, Entomology and Microbiology, Iowa State University, Ames, IA, USA; Department of Plant Pathology, Entomology and Microbiology, Iowa State University, Ames, IA, USA

**Keywords:** *Diabrotica virgifera virgifera*, greenhouse bioassay, insecticide screening, maize

## Abstract

Western corn rootworm, *Diabrotica virgifera virgifera* LeConte, is a serious pest of maize (*Zea mays* L.) in the United States. Because western corn rootworm larvae live in the soil, conducting an on-plant bioassay to screen novel management tools can be challenging. This study aimed to identify growth media for a greenhouse bioassay system suitable for studying interactions between western corn rootworm larvae and maize plants. We assessed the effects of growth medium on the growth of maize plants and on the survival and development of western corn rootworm larvae. Additionally, we characterized how larval density affected rootworm survival in the bioassay system. Plants grew well in soil collected from an agricultural field; however, this bioassay environment also resulted in poor survival of western corn rootworm larvae. By contrast, larval survival was greatest when plants grew in vermiculite, but this medium tended to produce the lowest values for metrics of plant growth. In general, a potting medium was conducive to both higher levels of larval survival and plant growth metrics. These results suggest that use of a potting medium or a mixture of soil collected from the field with other amendments, such as potting medium, could provide an environment conducive to both the growth of maize plants and the survival of rootworm larvae. This bioassay approach offers a novel bioassay system, which may potentially be applied to screen insecticides, microbial biopesticides, and plant-incorporated protectants.

## Introduction

Western corn rootworm, *Diabrotica virgifera virgifera* LeConte, is a serious maize (*Zea mays* L.) pest in North America and, along with other *Diabrotica* species, can impose economic loss as great as 2 billion dollars annually (Wechsler and Smith 2018). The life cycle of this univoltine pest is tightly linked to the phenology of maize, which is its only agricultural host (Spencer et al. 2009). Eggs hatch in late spring, and larvae feed on maize roots before pupating in the soil. Adults begin emerging from the soil late spring to summer, feeding on maize silks and pollen and depositing eggs in the soil of maize fields (Spencer et al. 2009). Eggs then enter an obligate diapause and overwinter in the soil (Spencer et al. 2009). Feeding on maize roots by western corn rootworm larvae can reduce the capacity of maize plants to acquire nutrients and water from the soil, thereby reducing yield (Spike and Tollefson 1991). This feeding can also weaken the plant, making it more susceptible to lodging, complicating harvest, and further reducing yield (Spike and Tollefson 1991, Godfrey et al. 1993, Gray and Steffey 1998).

Long-term, sustainable management of insect pests of economic importance, such as western corn rootworm, requires the use of integrated pest management (IPM) (Crowder et al. 2006, Onstad et al. 2014). The principles of IPM include monitoring pests and using appropriate management tactics at the appropriate time, which may consist of insecticides, plant-incorporated protectants, biological control, and biopesticides, among other tactics (United States Environmental Protection Agency 2023). Historically, western corn rootworms have been managed with the use of soil-applied insecticides to kill larvae, foliar insecticides to kill adults and prevent egg laying, and crop rotation (Levine and Oloumi-Sadeghi 1991, Meinke et al. 2021). The emergence of plant-incorporated protectants, such as genetically engineered maize that produces insecticidal proteins derived from the bacterium *Bacillus thuringiensis* (Bt), introduced another tactic that has been widely adopted in the United States, with roughly 85% of maize planted in the United States in 2023 producing one or more Bt toxins (United States Department of Agriculture, Economic Research Services 2023). To date, western corn rootworm populations in various regions of the United States have evolved resistance to Bt maize (Gassmann et al. 2011, 2014, 2016, Wangila et al. 2015, Zukoff et al. 2016, Ludwick et al. 2017, Gassmann 2021, Reinders et al. 2022), crop rotation (Levine et al. 2002), and insecticides used for management of adults (Meinke et al. 2021), highlighting the need to identify additional options that can be used to manage this serious pest of maize.

Bioassays offer 1 approach to screen for biological activity of management tools, which is an important first step in identifying a successful practice that might be added to an IPM framework (Gokulakrishnaa and Thirunavukkarasu 2023). Although bioassays are unable to account for all conditions that might be experienced in the field, they can begin to characterize the important interactions between a pest and a management tool. For example, western corn rootworm laboratory bioassays have been utilized to assess the effectiveness of Bt maize (Vaughn et al. 2005, Wen and Chen 2018, Gassmann et al. 2025). Additionally, bioassays have been used to assess the effectiveness of insecticidal compounds (Owens et al. 1974, Wright et al. 2000, St Clair et al. 2020) and microbial biopesticides (Hoffmann et al. 2014, Geisert et al. 2023). For a soil-borne pest such as larval western corn rootworm, a necessary step is to assess the impact of the soil environment on the efficacy of any management agent. Here, we report the results of experiments aimed at identifying growth media that might be used in an on-plant, greenhouse-based bioassay methodology that might be used to test various management tactics targeting western corn rootworm larvae.

## Methods

The aim of this study was to conduct a series of experiments to test the effects of growth medium and larval density on the growth of maize plants and the survival and development of western corn rootworm larvae with the goal of developing a bioassay that could be used to screen various insecticidal agents, including soil-applied conventional insecticides and microbial biopesticides, and plant-incorporated protectants. Experiments were conducted under greenhouse conditions between June and November 2022. Our initial experiments measured the effects of medium type on the growth of maize plants in our bioassay system. A follow-up experiment tested the effect of larval density on larval development and survival, and on the growth of maize plants. A final capstone experiment evaluated the effects of medium type on larval survival, larval developmental rate, and the growth of maize plants in the presence of rootworm larvae. To minimize the effect of environmental conditions over time, each experiment was structured as a randomized complete block design, with each treatment randomly assigned within a block, and one block planted weekly.

### Initial Experiments Comparing Growth Media

Two experiments were conducted to test the effects of growth media on maize plants. In all cases, plants were grown in a greenhouse using small cylindrical containers (3.8 cm diameter×18.4 cm length; volume = 158 ml) (Cone-tainers; SC10—Ray Leach “Super Cell” Air Pruning, Stuewe & Sons Inc, Tangent, Oregon). The first experiment consisted of 4 growth media: Vermiculite (VWR International, Radnor, Pennsylvania), crushed field soil collected in the spring from an agricultural field which had previously been planted to soybean in Story County, Iowa (34.6% clay, 50.5% silt, 15% sand, 3.6% soil organic matter, pH 7.5), potting medium (Berger BM7, Berger, Sheboygan, Wisconsin), and a mixture of 50% field soil and 50% potting medium (ie 1:1 mixture). The field soil was prepared for bioassays by air drying, mechanically crushing, and then passing through a screen to obtain a maximum particle size of <1 mm. In this experiment, there were 8 plants per medium type per block, and a total of 8 blocks were run, which equates to 64 plants per medium type and 256 plants for the entire experiment.

In the second experiment, 2 media were tested, which had not been evaluated in the first experiment. First was a 3-part mixture composed of equal amounts, by volume, of perlite (Sta-Green Organic Perlite, IMC Outdoor Living, Des Peres, Missouri), sand (Quikrete All-Purpose Sand, Quikrete International, Inc., Atlanta, Georgia), and vermiculite. The second medium was a 4-part mixture composed of equal parts, by volume, of field soil, perlite, sand, and vermiculite. There were 8 plants per medium type per block, and a total of 9 blocks were run, which equates to 72 plants per medium type and 144 plants for the entire experiment.

**Fig. 1.**
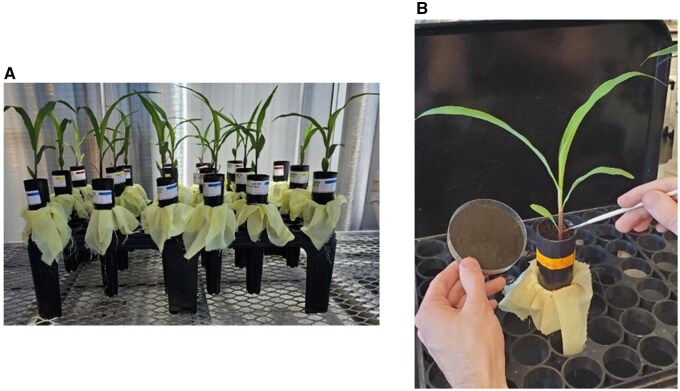
Photographs depicting the bioassay method tested in this study: A) plants in bioassay containers arranged on a greenhouse bench and B) process of adding neonate western corn rootworm larvae to maize plants.

To conduct bioassays, cylindrical containers were placed one inside a second, with a 12 ×12 cm^2^ piece of mesh cloth (Poly Chiffon, Hobby Lobby Stores Inc., Oklahoma City, Oklahoma) placed in between the containers to prevent loss of the growth medium through drainage ports (Fig. 1A). The top container was filled with 100 ml of growth medium (approximately 3-quarters full), lightly compressed, and then filled to the top with additional growth medium, after which each container was watered until the medium was fully saturated and water flowed freely from the bottom.

A cylindrical opening, approximately 2.5 cm deep, was made in the growth medium at the center of each container, and a single maize seed (lacking any pesticidal seed treatment) was added. Maize seeds (Viking U42-92, Albert Lea, Minnesota) were planted singly in containers and covered by pinching the medium around the opening and then lightly compacting the medium. Plants were randomized and kept in the greenhouse (16:8 h light/dark) with supplemental illumination provided by 1,000 W high-pressure sodium lights (PL3000, P.L. Light Systems, Hamilton, Ontario, Canada). Once seedlings emerged, they were checked daily and watered as needed in 50 ml increments. This was done because of the varying water-holding capacity of the growth media, with supplemental water provided when the top 5 cm of the growth medium was dry. For example, seedlings grown in vermiculite required watering daily, while those grown in field soil required water twice a week. Fourteen days after planting, each plant received 20 ml of a fertilizer solution (2.82 g/l of water, Miracle-Gro Soluble All-Purpose Plant Food, Scotts Miracle-Gro Company, Marysville, Ohio).

Twenty days after planting, measurements were taken on plant height, basal diameter, number of fully developed leaves, and leaf color. A leaf was defined as fully developed if it had a visible collar at its base (Abendroth et al. 2011). Plant height was measured from the base of the stem to the tip of the tallest leaf when extended, and leaf color was scored on a 1 to 4 color scale: 1 = pale yellow, 2 = yellow, 3 = light green/striped, and 4 = dark green.

At 21 d after planting, the aboveground biomass was removed at the soil line and placed, individually by plant, in an envelope that was then held at 60 °C for 2 wk in a drying oven (Thermo Scientific, Marietta, Ohio). Belowground biomass, including growth medium, was removed from the containers, and the growth medium was removed by rinsing the roots over a No.5-sized sieve (openings = 4.00 mm) stacked on top of a No. 30-sized sieve (openings = 600 µm) (Hogentogler & Co., Inc., Columbia, Maryland). Sieves were used to prevent the loss of root tissue while washing away the growth medium. Once the growth medium was removed, roots were placed, individually by plant, in small envelopes and held in a drying oven for 2 wk at 60 °C. After envelopes were removed from the drying oven, samples were allowed to equilibrate to ambient laboratory conditions for at least 1 wk before measuring mass. Aboveground and belowground biomass for each plant was measured to the nearest 0.1 mg with an analytical balance (Mettler Toledo XS205 Dual Range Balance, Switzerland).

### Larval Density Experiment

We conducted this experiment to test how larval density affected the survival and development of western corn rootworm larvae on maize plants in our bioassay system. The western corn rootworm strain used in this study was a non-diapausing laboratory strain, described in (Branson 1976), and was obtained from the United States Department of Agriculture (USDA), Agricultural Research Services (ARS), North Central Agricultural Research Laboratory in 2009. Since obtaining this strain from USDA ARS, the strain has been in continuous culture at Iowa State University following methods described in Deitloff et al. (2016). Western corn rootworm eggs were collected in Petri dishes containing finely sieved field soil (particle size<0.177 mm) over a period of 3 to 4 d. After collecting eggs, the contents of the Petri dishes were placed on a No. 30-sized sieve (openings = 600 µm) set atop a No. 60-sized sieve (openings=250 µm) (Hogentogler & Co., Inc., Columbia, MD) to separate eggs from soil particles. Eggs were then placed onto sieved field soil which had not been previously used for oviposition, with roughly 5,000 eggs per Petri dish, and then covered with finely sieved field soil and lightly moistened with deionized water. Petri dishes were sealed with parafilm and placed in an environmental chamber (26 °C; 76% R.H.; 0:24 h light/dark) (Percival, Perry, Iowa) until larvae eclosed. Newly eclosed neonates (<24 h old) were added to plants at densities of 0, 8, 13, and 20 larvae per plant, for a total of 4 treatments.

Cylindrical growing containers received a growth medium of a 1:1 mixture of potting medium (Berger BM7) and dried and crushed field soil. Maize seeds were planted singly in moistened growth medium and allowed to grow in a greenhouse, with supplemental lighting (16:8 h light/dark), for 13 d, receiving water as needed. Plants received 20 ml of fertilizer solution (2.82 g/liter of water, Miracle-Gro Soluble All-Purpose Plant Food) 7 and 13 d after planting. On day 14, the growth medium was gently moved to expose the uppermost roots, and neonate western corn rootworm larvae were placed on the roots using a fine-point paintbrush (Silver Bristlon 1900 round, Silver Brush Limited, Windsor, New Jersey) (Fig. 1B). Then, the soil was gently replaced. Plants did not receive water in the first 24 h after larvae were added, and then they were watered as needed through the end of the experiment.

On day 21, data were collected for plant height, basal diameter, number of fully developed leaves, and leaf color. Then, the aboveground biomass was removed, placed in small envelopes, and held in the drying oven for 14 d at 60 °C. The roots and growth medium with larvae were placed on a Berlese funnel for 3 d to extract any living larvae into glass vials containing 85% ethanol. Larvae were then counted using a stereo microscope (Leica Microsystems, Wetzlar, Germany), and larval head capsule width was measured using a stereo microscope with a digital camera and associated image analyzing software (Moticam 2500, Motic Images Plus 3.0; Kowloon, Hong Kong, China). Larval instar was determined based on head capsule width following Hammack et al. (2003).

In this experiment, there were eight plants per density per block, and a total of nine blocks, which equates to 72 plants per density and a total of 288 plants among the 4 densities tested. Data on plant metrics were not collected during the first block, with the exception of dry mass, which was collected for all nine blocks.

### Capstone Experiment

Based on the results of the initial study comparing growth media and the larval density study, a follow-up capstone experiment was performed to quantify western corn rootworm survival and development, and plant growth, across 6 media: vermiculite, field soil, potting medium, a 1:1 mixture of field soil and potting medium, a 3-part mixture of perlite, sand, and vermiculite, and a 4-part mixture of field soil, perlite, sand, and vermiculite. Based on the results of the larval density study, 16 neonate western corn rootworm larvae were placed on each plant in this study. Two plants per medium type were grown per block, with a total of 8 blocks. In total, 16 plants were tested for each medium type, and 96 plants were tested for the entire experiment.

As with the other experiments, plants were grown in a greenhouse, with supplemental lighting (16:8 h light/dark), and methods for growing plants were the same as those used in the other experiments. Plants were fertilized 7 and 12 d after planting. Fourteen days after planting, growth medium was gently moved to expose the roots of maize plants in bioassay containers, and 16 neonate larvae (< 24 h old) were placed onto the roots as described in the larval density experiment.

On day 21, data were collected for plant height, basal diameter, number of fully developed leaves, and leaf color. At that time, the aboveground biomass was removed, placed in envelopes, and held in a drying oven. Roots and medium, along with rootworm larvae, were removed from the cylindrical containers and placed on Berlese funnels for 3 d to extract surviving larvae into vials of 85%ethanol. Larvae were counted using a stereo microscope and scored for larval instar following Hammack et al. (2003).

### Data Analysis

Data were analyzed in SAS 9.4 (SAS Institute Inc., Cary, North Carolina). Data on plant metrics for the experiments comparing media, including plant height, basal diameter, aboveground biomass, and belowground biomass, were each analyzed with a mixed-model, one-way analysis of variance (ANOVA) (PROC MIXED). The fixed factor in the model was medium type, and random factors were block and block by medium type. Root-to-shoot ratio of dry mass was calculated as the quotient of belowground biomass divided by aboveground biomass. Data on root-to-shoot ratio were log10 transformed to ensure the normality of the residuals and analyzed with a mixed-model, one-way ANOVA. For both leaf number and leaf color score, residuals did not follow a normal distribution and consequently means were compared using a non-parametric Kruskal-Wallis test (PROC NPAR1WAY). To avoid pseudoreplication, data for each treatment were averaged within each block before analysis.

For the larval density experiment and capstone experiment, the plant metrics of aboveground biomass, basal diameter, and height were analyzed with a mixed-model, one-way ANOVA (PROC MIXED). In the density experiment, the fixed factor was larval density, and random factors were block and block by larval density, while in the capstone experiment the fixed factor was the medium type, and random factors were block and block by medium type. Data on the plant metrics of leaf number and color score were analyzed with a Kruskal–Wallis test (PROC NPAR1WAY), with data for each treatment averaged within blocks prior to analysis. Proportion larval survival was calculated as the quotient of the number of larvae collected from a plant divided by the number of larvae initially placed on that plant. Additionally, for both experiments, the proportion of third-instar larvae (ie final larval instar) was calculated as the quotient of the number of third-instar larvae collected per plant divided by the total number of larvae recovered from that plant. The proportion of surviving larvae and the proportion of third-instar larvae were transformed by the arcsine of the square root to ensure normality of the residuals, and the results were analyzed with a mixed-model one-way ANOVA. In the density experiment, the fixed factor was density, and in the capstone experiment, the fixed factor was medium type. In both analyses, random factors were block and the interaction of block with the fixed factor. For all data analyzed with an ANOVA in these experiments, pairwise comparisons were conducted using PDIFF in PROC MIXED, with Tukey adjustment applied to account for multiple comparisons and a significance level of *P* <0.05.

## Results

### Initial Experiments Comparing Growth Media

**Fig. 2.**
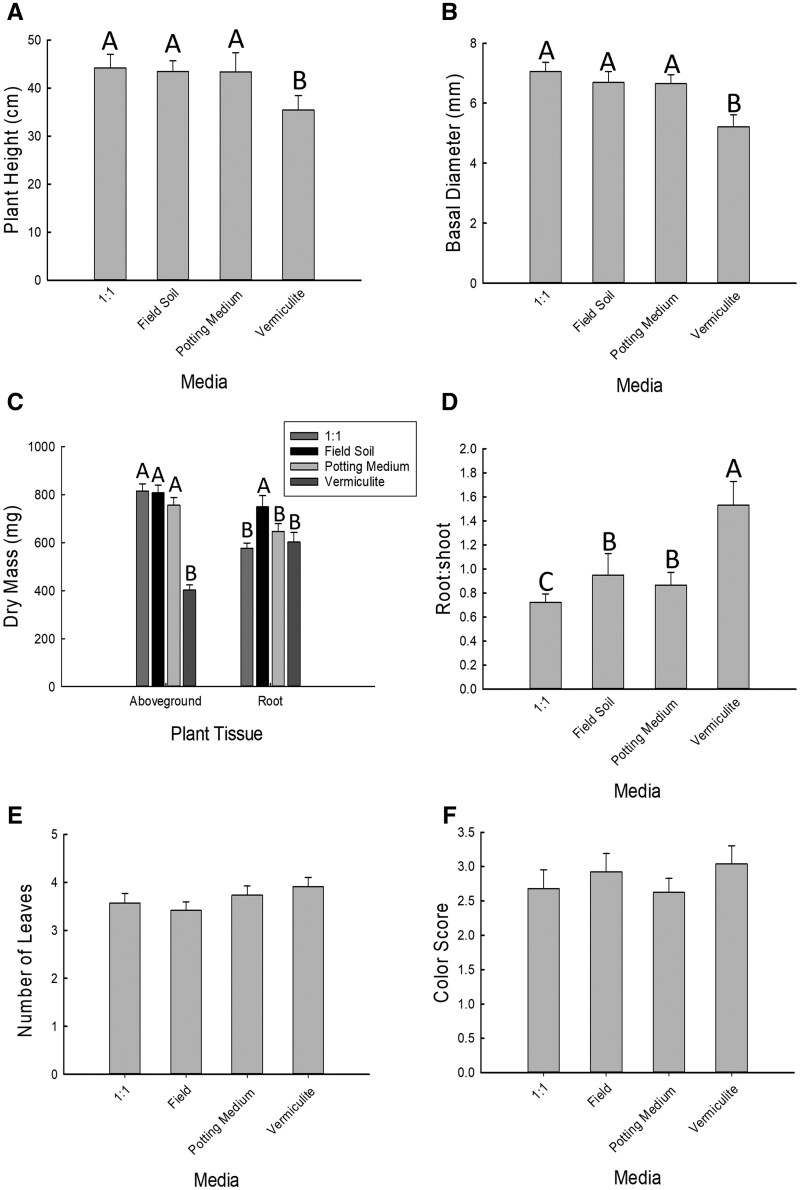
Effects of 4 growth media on plant A) plant height, B) basal diameter, C) dry mass, D) root-to-shoot ratio E) number of leaves, and F) color score. The media in which maize plants were grown included: **1:1**) a mix of equal parts field soil and potting medium, **Field**) pure field soil, which had been dried, crushed and sieved, **Potting Medium**) pure potting medium, and **Vermiculite**) pure vermiculite. Bars are sample means of 8 replicates for each treatment and error bars are the standard error of the mean. Letters indicate significant pairwise differences between means (Tukey adjustment, *P *< 0.05).

For all the variables measured in the first experiment comparing growth media, except for the belowground biomass, we observed a significant effect of growth medium (Table 1). Plants grown in vermiculite were significantly shorter than plants grown in field soil, potting medium, and a 1:1 mixture of field soil and potting medium, and had significantly smaller basal diameter and significantly lower aboveground biomass (Table 1; Figs 2A, B, and C). Plants grown in field soil had significantly greater belowground biomass than plants grown in either the 1:1 mixture of field soil and potting medium or vermiculite, with plants grown in potting medium having an intermediate level of belowground biomass (Table 1; Fig. 2C). The root-to-shoot ratio for plants grown in vermiculite was significantly greater than all other treatments, while the 1:1 mixture of field soil and potting medium had a significantly lower root-to-shoot ratio than treatments of only potting medium or field soil (Table 1, Fig. 2D). There was no significant effect of media type on the number of leaves (df = 3, χ^2^=5.54, *P* = 0.14) or leaf color score (df = 3, χ^2^=3.69, *P* = 0.30) (Figs 2E and 2F).

**Table 1.** Analysis of variance for maize plant metrics in the first growth media experiment

Measurement	df	*F*-statistic	*P*
Plant height	3, 15	4.44	0.02
Basal diameter	3, 15	10.7	<0.001
Aboveground biomass	3, 15	13.4	<0.001
Belowground biomass	3, 15	1.32	0.30
Root-to-shoot	3, 15	12.6	<0.001

Growth media used in the experiment were vermiculite, field soil, potting medium, and a 1:1 mixture of equal parts field soil and potting medium.

**Fig. 3.**
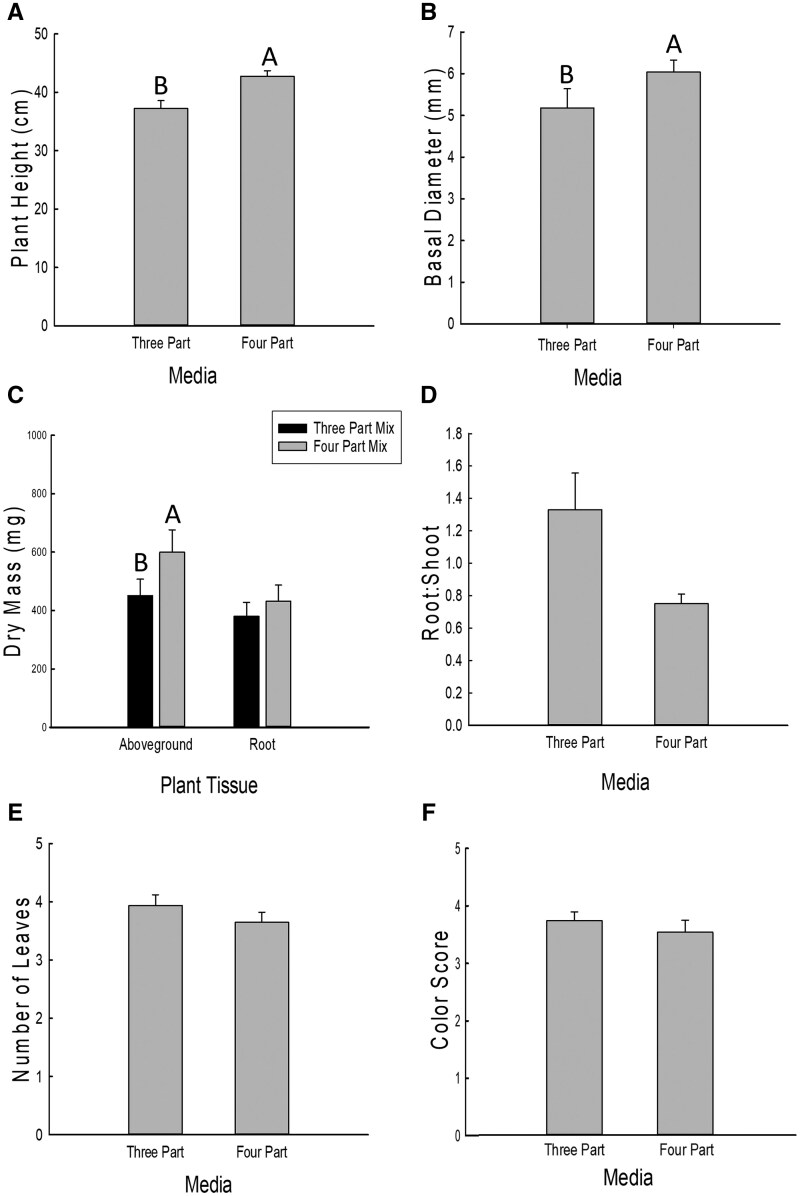
Effects of two growth media on plant A) height, B) basal diameter, C) dry mass, D) root-to-shoot ratio, E) number of leaves, and F) color score. The media in which maize plants were grown and these included: **3 Part**) a 3-part mixture of perlite, sand, and vermiculite, and **4-Part**) a 4-part mixture of field soil, perlite, sand and vermiculite. Bars are sample means of 9 replicates for each treatment and error bars are the standard error of the mean (Tukey adjustment, *P *< 0.05).

In the second experiment comparing growth media, plant height was significantly greater for plants grown in the 4-part mixture (field soil, sand, perlite, and vermiculite) compared to the 3-part mixture (sand, perlite, and vermiculite) while basal diameter showed a trend to be greater in the 4-part mixture (Table 2, Figs 3A and 3B). Plants from the 4-part mixture had significantly more aboveground biomass than the 3-part mixture (Table 2, Fig. 3C). However, belowground biomass was not significantly different between the 2 media types (Table 2, Fig. 3C). There was a strong trend (*P* = 0.06) of higher root-to-shoot ratio for plants grown in the 3-part mixture than in the 4-part mixture (Table 2, Fig. 3D). Leaf number (df = 1, χ^2^=0.89, *P* = 0.35) and leaf color score (df = 1, χ^2^=0.95, *P* = 0.33) did not differ significantly between the 2 media types (Fig. 3E and 3F).

**Table 2.** Analysis of variance for maize plant metrics in the second growth media experiment

Measurement	df	*F*-statistic	*P*
Plant height	1, 7	21.6	0.006
Basal diameter	1, 7	5.98	0.058
Aboveground biomass	1, 7	20.7	0.003
Belowground biomass	1, 7	2.82	0.14
Root-to-shoot	1, 7	4.93	0.062

Growth media used in the experiment were 1) equal parts perlite, sand, and vermiculite and 2) equal parts field soil, perlite, sand, and vermiculite.

### Larval Density Experiment

Proportion larval survival and proportion of third-instar larvae did not differ significantly among density treatments, although there was a trend of a lower proportion of third instar larvae at the highest density compared with the other two densities (Tables 3 and 4). Plant measurements of aboveground biomass, height, basal diameter, number of leaves (df = 3, χ^2^=0.45, *P* = 0.93) and leaf color (df = 3, χ^2^=0.62, *P* = 0.89) were not significantly different across larval densities (Tables 3 and 4).

**Table 3.** Analysis of variance for larval and maize plant metrics in the western corn rootworm density experiment

Measurement	df	*F*-statistic	*P*
Proportion survival^a^	2, 16	1.78	0.20
Proportion third instar^a^	2, 16	1.78	0.20
Plant height^b^	3, 21	2.27	0.11
Basal diameter^b^	3, 21	0.920	0.45
Aboveground biomass^b^	3, 24	1.63	0.21

a The fixed factor in the analysis was the three density treatments of 8, 13, and 20 larvae per plant.

b The fixed factor in the analysis was the four density treatments of 0, 8, 13, and 20 larvae per plant.

**Table 4.** Sample means and standard errors for western corn rootworm larval and maize plant metrics in the larval density experiment

Measurement	Larval density^a^	Mean	SE
Proportion survival^b^	8	0.375	0.0750
	13	0.416	0.0702
	20	0.429	0.0748
Proportion third instar^c^	8	0.360	0.137
	13	0.378	0.122
	20	0.215	0.0970
Plant height (cm)	0	46.7	3.16
	8	48.0	2.51
	13	46.3	2.71
	20	46.5	2.74
Basal diameter (mm)	0	5.24	0.468
	8	5.33	0.428
	13	5.37	0.428
	20	5.33	0.400
Number of leaves	0	2.35	0.182
	8	2.34	0.204
	13	2.43	0.197
	20	2.33	0.202
Color score^d^	0	2.68	0.219
	8	2.71	0.208
	13	2.72	0.203
	20	2.58	0.208
Aboveground biomass (mg)	0	625	78.9
	8	646	73.7
	13	675	82.1
	20	686	87.2

a Larval density represents the density of larvae tested per plant: 0, 8, 13, and 20 larvae.

b Proportion survival represents the quotient of live larvae at the end of the assay divided by the number of larvae placed on the plant at the start of the assay.

c Proportion third Instar is the quotient of the number of third instar larvae recovered from a plant divided by the total number of larvae recovered.

d Color score is a numeric value assigned to represent the color of plants on a scale of 1 to 4.

### Capstone Experiment

**Fig. 4.**
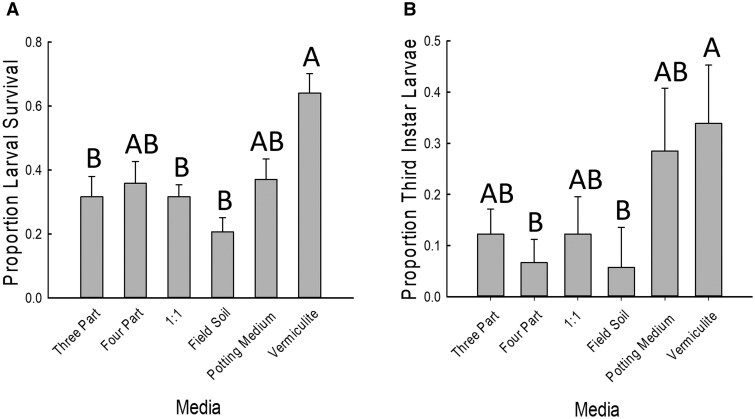
Effects of 6 growth media on A) larval survival and B) proportion of third instar larvae for western corn rootworm. The media in which maize plants were grown included: **3 Part**) a 3-part mixture of equal parts perlite, sand, and vermiculite, **4 Part**) a 4-part mixture of equal parts field soil, perlite, sand, and vermiculite, **1:1**) a mixture of equal parts field soil and potting medium, **Field Soil**) pure field soil, which had been dried, crushed, and sieved, **Potting Medium**) pure potting medium, and **Vermiculite**) pure vermiculite. Bars are sample means of 8 replicates for each treatment, and error bars are the standard error of the mean. Letters indicate significant pairwise differences between means (Tukey adjustment, *P *< 0.05).

Proportion larval survival differed significantly among growth media (Fig. 4A; Table 5). Larval survival was significantly higher in vermiculite than in field soil alone, the 1:1 mixture of field soil and potting medium, or the 3-part mixture of sand, perlite, and vermiculite. Survival in the 4-part mixture of potting medium, sand, perlite, and vermiculite and in potting media alone was intermediate and not significantly different from any of the treatments. The proportion of third instar larvae was also significantly higher in the vermiculite than in either the field soil or the 4-part mixture (Fig. 4B; Table 5).

**Table 5.** Analysis of variance for the effect of growth media on survival and development of western corn rootworm larvae and maize plant metrics in the capstone experiment^a^

Measurement	df	*F*-statistic	*P*
Proportion survival^b^	5, 35	4.63	0.002
Proportion third Instar^c^	5, 35	4.26	0.004
Plant height	5, 35	19.1	< 0.001
Basal diameter	5, 35	6.16	< 0.001
Aboveground biomass	5, 35	12.6	< 0.001

a Six types of plant growth medium were tested in this experiment: vermiculite; field soil; potting medium; a 1:1 mixture of field soil and potting medium; equal parts perlite, sand, and vermiculite; and equal parts field soil, perlite, sand, and vermiculite.

b Proportion Survival represents the quotient of live larvae at the end of the assay divided by the number of larvae placed on the plant at the start of the assay.

c Proportion third Instar is the quotient of the number of third instar larvae recovered from a plant divided by the total number of larvae recovered.

**Fig. 5.**
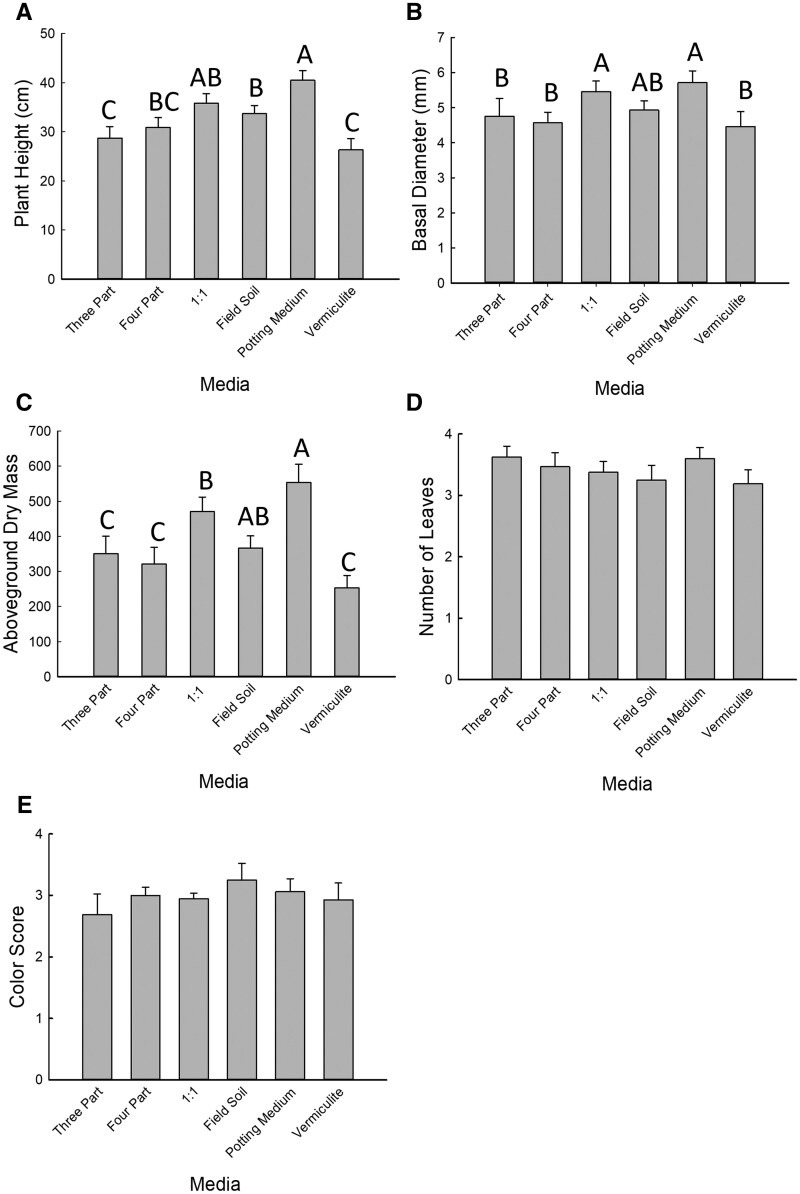
Effects of 6 growth media on plant A) height, B) basal diameter, C) dry mass, D) number of leaves, and E) color score. The media in which maize plants were grown included: **3 Part**) a 3-part mixture of equal parts perlite, sand, and vermiculite, **4 Part**) a 4-part mixture of equal parts field soil, perlite, sand, and vermiculite, **1:1**) a mixture of equal parts field soil and potting medium, **Field Soil**) pure field soil, which had been dried, crushed and sieved, **Potting Medium**) pure potting medium, and **Vermiculite**) pure vermiculite. Bars are sample means of 8 replicates for each treatment and error bars are the standard error of the mean. Letters indicate significant pairwise differences between means (Tukey adjustment, *P *< 0.05).

Significant effects of growth media were detected for plant height, basal diameter, and aboveground biomass (Table 5). The height of plants grown in potting medium was greater than any of the other media, with the exception of the 1:1 mixture of field soil and potting medium (Fig. 5A; Table 5). Plant height was lowest for plants grown in vermiculite and the 3-part mixture (Fig. 5A; Table 5). Basal diameter was significantly greater for plants grown in either potting medium or a 1:1 mixture of field soil and potting medium compared to any other treatment with the exception of field soil (Fig. 5B; Table 5). Aboveground biomass of plants grown in vermiculite was significantly lower than that grown in the 1:1 mixture or potting medium. Plants grown in potting medium also had significantly more aboveground biomass than any other growth medium (Fig. 5C). Leaf number (df = 5, χ^2^=4.12, *P* = 0.53) and leaf color score (df = 5, χ^2^=1.56, *P* = 0.91) did not differ significantly among treatments (Fig. 5D and 5E).

## Discussion

The goal of this study was to identify growth media for a greenhouse-based on-plant bioassay for larval western corn rootworm. Our results indicate that field soil tended to produce among the highest values for plant growth metrics but also had the lowest larval survival. By contrast, plants grown in vermiculite tended to have the lowest values for several plant growth metrics, but the greatest larval survival and development to third instar. We hypothesize that the field soil created a compacted (ie dense) environment, especially compared to the vermiculite, and this effect reduced larval survival in the field soil (Ellsbury et al. 1994). It is important to note that a single soil type was assessed in this study and factors such as soil composition, structure, and aggregate size should be considered before implementing field soil into any assay system. To address these issues, future bioassays could use several soil types to better assess effects across a range of soil properties such as texture, pH, and microbial community. Media containing a mixture of field soil with other amendments, or using potting medium alone, worked well for both plant growth and western corn rootworm larval survival. However, choosing an appropriate growth medium for a bioassay will depend on what is being tested, such as microbial biopesticides versus plant-incorporated protectants, or soil-applied insecticide.

In the density experiment, there was no significant difference in larval survival or development for the treatments of 8, 13, or 20 larvae applied to plants grown in a 1:1 ratio of field soil and potting medium. While not statistically significant, there was, however, a trend toward fewer third-instar larvae for plants that received 20 neonate larvae. This experiment aimed to identify how many western corn rootworm larvae can be supported on a plant without adverse effects of density-dependent mortality. In general, a higher number of larvae per bioassay could be advantageous because it may enable a researcher to better discern differences among treatments. However, past studies have found that western corn rootworm larvae often demonstrate a pronounced effect of density-dependent mortality, which can confound estimates of the actual level of mortality imposed by an insecticidal treatment (eg dose calculation for a transgenic crop) (Hibbard et al. 2010, McCulloch and Gassmann 2024). Gassmann et al. (2011, 2014, 2016) conducted studies in an environmental chamber using 12 larvae per plant, similar to our study, which used 16 larvae per plant in the capstone experiment. Larval density did not appear to affect plant growth in this study (Tables 3 and 4), suggesting that future studies could consider using larval densities greater than those evaluated here; however, significant reductions in larval growth rate, and potentially proportion survival, may arise at those higher densities.

Previous greenhouse studies have been conducted for western corn rootworm using field soil mixed with another substrate such as perlite or vermiculite to assess the effects of larval feeding on plant physiology, efficacy of insecticides, and larval development on a variety of host plants (Branson 1976, Owens et al. 1974, Riedell 1989). Application of insecticides can be incorporated into our bioassay system by mixing them into the growth medium before planting. This methodology has been demonstrated in laboratory assays by Rudeen et al. (2013) and St Clair et al. (2020), who incorporated conidia of insect pathogenic fungi and chemical insecticides, respectively, into 30 g of soil to assess the efficacy of their treatments against western corn rootworm larvae. Soil physical properties such as structure and texture should be considered carefully before use in an assay system as they can shape the interactions occurring in the soil, including the development of plant roots, microbial community composition, and movement of water and nutrients through the soil matrix (Brady 1984, Barbercheck 1992). While soil properties such as soil organic matter content and the microbial community may be conserved in the field-collected soil used for greenhouse assays, mechanical disturbance (ie collection and handling) of field soil may disrupt soil bulk density and reduce pore space leading to a compact environment as likely arose in our media and capstone experiments.

The microbial community present in a plant’s growth medium can affect plant growth, plant-insect interactions, and interactions between soil-borne insects and microbial biopesticides, including insect pathogenic fungi, and nematodes (Jaronski 2007, Dematheis et al. 2013, Blum 2014). Dematheis et al. (2013) demonstrated that the presence of an arbuscular mycorrhizal fungus, a plant-benefiting endophyte, led to a reduction in western corn rootworm larval development. Multitrophic studies such as this could be evaluated using our bioassay system to assess the effect of different soil microbial communities on establishment of beneficial fungi. In the case of entomopathogenic fungi or nematodes, adding field soil to the bioassay system could provide a better understanding of the microbial community’s influence on insecticidal activity (Hassani et al. 2018). Soil texture is also an essential factor that can influence pests’ movement through the soil and, therefore, the probability of interacting with a microbial biopesticide (Jaronski 2010). Results from our capstone experiment show that field soil alone resulted in lower larval survival and third instar development. As such, a growth medium that might work well for assays with microbial biopesticides could consist of a mixture of field soil and potting medium. However, many of the entomopathogenic fungi used as biopesticides are known to grow saprophytically (Goettel and Inglis 1997) and may benefit from the high level of organic matter in a potting medium, leading to levels of fungal density that would not be found in field soil. Therefore, a mixture of field soil with other amendments such as perlite or vermiculite might be more appropriate.

The soil environment is also an important factor in the movement, sorption, and degradation of chemical insecticides (­Wauchope et al. 2002). Like microbial biopesticides, the efficacy and persistence of chemical insecticides are influenced by both biotic and abiotic conditions. Bacterial and fungal communities in soils are capable of degrading conventional insecticides (Gilani et al. 2016). Additionally, soil properties such as pH, percentage of organic matter, and soil moisture can influence the sorption rate of the insecticidal compounds, which can affect the persistence and activity of the insecticides against target pests (Wauchope et al. 2002, Liu et al. 2018). Therefore, bioassays that use a growth medium containing field soil to screen chemical insecticides may better represent efficacy in the field, however, additional amendments to field soil should be considered to enhance survival of the rootworm larvae.

In the case of plant-incorporated protectants such as transgenic maize plants that produce insecticidal proteins derived from the bacterium Bt, ingestion of the plant tissue is necessary to cause insect mortality. Production of insecticidal proteins is not expected to be strongly influenced by the soil microbial community (Parker and Sander 2017). In this case, a potting medium, which is suitable for maize growth and promotes larval movement through the substrate and larval feeding on maize roots, may be well suited to characterize the effect of the plant-incorporated protectant on larval mortality. This is supported by multiple studies that used potting medium to assess Bt traits against western corn rootworm populations (Gassmann et al. 2011, 2014, 2016, Wangila et al. 2015, Reinders et al. 2018).

The bioassay method developed here also has the potential to be applied to test management tactics against other root-feeding pests (Johnson et al. 2016). For example, wireworms (Coleoptera: Elateridae), which consist of multiple polyphagous species, are pests of concern for several crops and could potentially be studied using this bioassay system and an annual crop such as maize or wheat (Furlan 1998, Vernon et al. 2008). Also, the study of root-feeding aphids might also be readily adapted to this bioassay system. While the focus of this study was to compare various bioassay approaches for western corn rootworm larvae on maize, the general methodology presented here could be modified to provide a high throughput assay for other root-feeding pests.

On-plant greenhouse assays are an important step to assess pesticide use before implementing them into a field trial to reduce the variability of environmental conditions such as water availability or the density of naturally occurring pest populations. Our research has established a high throughput assay that can use several variations of growth media to expand the type of insecticides being tested, including microbial biopesticides. Additionally, this system could be used to test compatibility between microbial biopesticides, conventional pesticides, and plant-incorporated protectants.
